# From being restrained to recapturing vitality: non-western immigrant women’s experiences of undergoing vitamin D treatment after childbirth

**DOI:** 10.1080/17482631.2019.1632111

**Published:** 2019-06-24

**Authors:** Ninni Qvist, Ingrid Bergström, Torbjörn Åkerstedt, Jan Persson, Hanne Konradsen, Anette Forss

**Affiliations:** a Osteoporosis Center, Inflammation & Infection Theme, Karolinska University Hospital Huddinge, Sweden; b Department of Clinical Science, Intervention and Technology, Karolinska Institute, Sweden; c Stress Research Institute, Stockholm University, Sweden; d Clinical Neuroscience, Karolinska Institute, Stockholm, Sweden; e Behavioral Medicine, Karolinska University Hospital, Stockholm, Sweden; f Department of Neurobiology, Care Sciences and Society, Division of Nursing, Karolinska Institute, Stockholm, Sweden; g Department of Philosophy, Stony Brook University, New York, USA

**Keywords:** Non-western immigrant women, low vitamin D levels, women’s health, post pregnancy, musculoskeletal pain, sleep, physical weakness, qualitative methods

## Abstract

**Purpose**: Vitamin D deficiency is a complex topic in human health and ill-health and has been studied in a variety of contexts and populations. Few studies examine Vitamin D deficiency among non-western immigrant women and even fewer examine women’s perspective on daily life while living with low vitamin D levels after childbirth and undergoing vitamin D treatment. The aim was, therefore, to explore health and ill-health among non-western immigrant women living with low vitamin D levels after childbirth and reaching normalized levels after one year of vitamin D treatment.

**Method**: An explorative qualitative study using qualitative content analysis. Six women aged 25 to 38 years, diagnosed with low 25-hydroxyvitamin D levels during pregnancy, were recruited after having undergone vitamin D treatment.

**Results**: The women told about living a restrained life which gradually transformed into an experience of recaptured vitality. They also experienced a need for continuity in medication, as an interruption of treatment meant returning symptoms.

**Conclusion**: In this study, non-western immigrant women described benefits in everyday life, increased strength, relieved pain and improved sleep quality. The findings can provide valuable knowledge for healthcare providers meeting women with physical weakness, musculoskeletal pain and/or poor sleep quality after childbirth. Further studies using a longitudinal design and larger samples are warranted.

## Introduction

Vitamin D deficiency is a complex topic in human health, and ill-health and has been studied in a variety of contexts and populations. Vitamin D levels can be measured by analysing 25-hydroxyvitamin D, in short; 25(OH)D, via a blood sample. Institute of Medicine (IOM) concludes that there is a lack of evidence-based consensus about levels of 25(OH)D regarding vitamin D deficiency and excess (Ross, Taylor, Yaktine, & Del Valle, 2011). The IOM report suggests that levels of 25(OH)D under 30 nmol/l are to be considered risk for deficiency, levels between 30 and 50 nmol/l can be inadequate and insufficient for some, but not everyone, while levels >50 nmol/l are considered sufficient (Ross, Taylor, Yaktine, & Del Valle, 2011, pp. 14–15). It is known that persistently low levels of 25(OH)D might lead to osteomalacia and severe symmetrical musculoskeletal pain in the lower back and lower extremities as well as muscular weakness (Reginato & Coquia, 2003). There have also been reports of patients with symmetrical musculoskeletal pain related to vitamin D deficiency but without evidence of osteomalacia (Gloth & Greenough, 2004). Pain is one of the reported symptoms of low levels of vitamin D. The association between musculoskeletal pain and vitamin D deficiency is well documented, especially in women (Al Faraj & Al Mutairi, 2003; Gloth & Greenough, 2004; Lotfi, Abdel-Nasser, Hamdy, Omran, & El-Rehany, 2007), and in immigrants in Europe and North America (de Torrenté de la Jara, Pécoud, & Favrat, 2004; Erkal et al., 2006; Helliwell, Ibrahim, Karim, Sokoll, & Johnson, 2006). Treatment with vitamin D has shown to provide pain relief in various groups of patients (Helde-Frankling & Björkhem-Bergman, 2017; Wepner et al., 2014; Wu, Malihi, Stewart, Lawes, & Scragg, 2016). However, a Cochrane Systematic Review found no correlation between vitamin D supplementation and pain reduction in patient with chronic pain (Straube, Derry, Straube, & Moore, 2015).

Few studies have examined the effects of vitamin D treatment on musculoskeletal pain among immigrant women with a vitamin D deficiency. Results have been contradictory, some studies have shown effects of treatment on musculoskeletal pain (Al Faraj & Al Mutairi, 2003; de Torrenté de la Jara et al., 2004; Englund, Persson, & Bergstrom, 2017), while other studies have not (Helliwell et al., 2006; Knutsen, Madar, Brekke et al., 2014; Sandoughi, Zakeri, Mirhosainee, Mohammadi, & Shahbakhsh, 2015; Schreuder, Bernsen, & van der Wouden, 2012). One study including a multi-ethnic population in Norway showed that daily supplementation of vitamin D did not reduce scores for musculoskeletal pain, although the prevalence of hypovitaminosis D among these patients was high (Knutsen, Brekke, Gjelstad, & Lagerløv, 2010). There are also only a few studies investigating the association between vitamin D status and muscle strength among these women, and the results for this symptom are contradictory as well. Two studies of young, Somali, immigrant women in Sweden found an association between vitamin D status and muscle strength (Kalliokoski, Bergqvist, & Löfvander, 2013; Kalliokoski, Rodhe, Bergqvist, & Löfvander, 2016). Also, a meta-analysis found substantially higher proximal muscle strength with vitamin D treatment in vitamin D-deficient individuals (Stockton, Mengersen, Paratz, Kandiah, & Bennell, 2011). However, there are also studies that found no effect of vitamin D treatment on muscular function in people with vitamin D deficiency (Goswami et al., 2012; Knutsen, Madar, Lagerløv et al., 2014). The association of vitamin D deficiency and fatigue has been sparsely studied; findings in studies on varied groups of patients are inconsistent, and there is no clear evidence that vitamin D reduces fatigue (Kampman, Steffensen, Mellgren, & Jørgensen, 2012; Ruiz-Irastorza, Gordo, Olivares, Egurbide, & Aguirre, 2010). An association between vitamin D deficiency and sleeping disorders has been found (Gao et al., 2018; McCarty, Reddy, Keigley, Kim, & Marino, 2012). Among non-western immigrants in the Nordic region, two factors contributing to a high prevalence of vitamin D deficiency are a darker skin tone and less solar exposure, for example, owing to traditional clothing covering much of the skin (Bergström, Palmér, Persson, & Blanck, 2014; Holvik, Meyer, Haug, & Brunvand, 2005).

A study was conducted finding a high prevalence of vitamin D deficiency in pregnant immigrant women living in Sweden (Bergström et al., 2014). The finding was followed by an intervention study which examined the treatment of vitamin D among immigrant women after childbirth, and found an effect on musculoskeletal pain and increased muscular strength (Englund et al., 2017). However, these quantitative studies did not provide knowledge about the women’s perspective on living with low vitamin D levels, receiving vitamin D treatment, and reaching normalized vitamin D levels, in daily life. We, therefore, decided to further explore this.

### Aim

The aim of this qualitative interview study was to investigate health and ill-health in daily life among non-western immigrant women living with low levels of vitamin D after childbirth and reaching normalized vitamin D levels after one year of vitamin D treatment.

## Method

A qualitative paradigm acknowledges that reality is complex, context-dependent, and has multiple meanings and that knowledge can be derived from participants in natural settings (Monti & Tingen, 2006). A qualitative exploratory design using open interviews was chosen, data were analyzed using content analysis.

### Participants

The women in this study were recruited from the above-mentioned intervention study (Englund et al., 2017). All women who reached sufficient vitamin D levels after one year of treatment with 800–1,600 IU cholecalciferol and 500–1,000 mg calcium as daily doses in the form of pills were asked to participate. One woman with levels just below (≥47 nmol/l) was also included. Exclusion criteria were previous vitamin D treatment, remaining low levels of vitamin D after treatment, and/or a new pregnancy. Twelve women were eligible for inclusion, two women had become pregnant, and one woman became vitamin D deficient again. The remaining nine women were invited to participate in the interview study. The women were informed of the study via a letter sent to their home. The letter also contained information about a coming telephone call from one of the researchers after approximately one week to pose possible questions about the study, and to report their decision on whether they wanted to participate or not. Three women declined participation. Six women accepted participation and were included in the study. The vitamin D levels of these six women varied between 17 and 36 s-25(OH) D prior to treatment, and between 47 and 74 s-25 (OH) D after one year of vitamin D treatment (see Table I).

**Table I.** Vitamin D (25(OH)D) levels.

	25(OH)D levels nmol/l	
Woman	Treatment start	After 1 year
Olivia	21	61
Rosa	32	58
Stella	17	61
Emma	28	74
Maria	36	47
Hanna	20	65

The ages of the six women participants were between 25 and 38 years (median age was 34.5 years). Five women were married, one was single. The women had one to three children, and their educational levels ranged from primary school to college. They originated from four different continents; Africa (n:2), South America (n:1), the Middle East (n:2), and Eastern Europe (n:1). All women were now living in suburban areas of Sweden.

### Data collection

All women were provided with the opportunity to choose the interview location. The interviewer was one of the authors (NQ). Three women preferred the interviewer´s workplace, and three others preferred to be interviewed at home. All interviews were digitally audio recorded with the women´s permission, lasting between 25 and 45 min, with an average of 32 min. The interviewer used a semi-structured interview guide with open questions to enable the women to describe daily life after childbirth while undergoing vitamin D treatment in their own words (Kvale & Brinkmann, 2009). All interviews started with an open question about how the women currently felt about their health, and they were then asked to describe an ordinary day and their experiences of being treated with vitamin D. Follow-up questions were asked for clarification based on their responses.

### Data analysis

Qualitative content analysis was chosen to analyze the interviews focusing on the manifest, discernible content of the interviews (Elo & Kyngäs, 2008; Graneheim, Lindgren, & Lundman, 2017; Graneheim & Lundman, 2004; Hsieh & Shannon, 2005). Authors NQ and AF conducted the analysis. Inductive content analysis was chosen, since this approach is recommended when there is little prior knowledge of the phenomenon being investigated (Elo & Kyngäs, 2008). All interviews were transcribed verbatim by the first author. Specific names and places that could lead to identification were excluded during transcription. At first, interviews were read several times for an overall sense of content. The text was then divided into meaning units, delineated by its content being contextually inter-linked (Graneheim & Lundman, 2004). The texts in the meaning units were condensed to shorten the text while maintaining its content. The condensed meaning units were then labelled with descriptive codes. Codes with similar content were grouped into categories. In the discussion of categories, the researchers all looked at data, and in case of inconsistencies, decisions were made in consensus to enhance validity as recommended by Elo and Kyngäs (2008).

The findings are presented along with quotes supporting the categories resulting from the analysis process.

### Ethical considerations

The Swedish regional ethics committee approved the study prior to initiation (number 2012/426–32). The information letter sent to all women participants described the purpose of the study that participation was voluntary, with the right to withdraw with no consequences for their continued care or contact with the healthcare system. Those willing to participate were asked to sign and return the pre-paid letter while keeping a personal copy. Thereafter, the first author contacted the participants and asked whether they had questions or preferences for the interview locations. The interviewer asked the women after the interview for any sensitive information they wished to be carefully dealt with during transcription. All interviews were de-identified during transcription, e.g., by omitting personal names, country of origin, and workplace.

## Findings

A process of living a restrained life prior to the discovery and treatment of their low levels of vitamin D (LLVD hereafter) were described. During the treatment, an increased physical strength and a recaptured vitality were present, while an instant backlash occurred when interrupting the medication.

### Living a restrained life

Descriptions of a paralyzing and inhibitory physical weakness during the period of LLVD were common among the women. Physical weakness affected everyday life, both at home and away, in a variety of ways. The weakness could limit the number of daily activities and decreased the physical strength to overcome daily chores at home. One woman described how weakness and tiredness meant that she had to prioritize what to do during the day: *Simply MOVING tired me out … I couldn´t do anything, I just didn’t have the strength … two things on the same day were too much for me (Olivia)*

The weak body inhibited ordinary activities in daily life, as it was unpossible to ’speed up’ when, for example, being in a hurry to catch the bus or being unable to come for a doctor’s appointment knowing that an invoice would come due to the missing visit.

All women described experiences of pain restricting and inhibiting their daily lives. For example, walking with the baby carriage became extremely slow due to pain in the legs and back. The pain could be constant, intense and felt in both muscles, bones, back, rib cage, legs, back of the knees, the knee joint and wrist. All this meant difficulties in stretching out, swimming, relaxing at bedtime, and doing pleasant things like dancing and exercising. Rosa described the pain in her whole body a long period after childbirth:

So if I sat, and tried to stand … it felt as if I had/ … /soreness, even though I hadn´t been exercising so much … I had severe pain throughout my body./ … /I don´t know what it´s like for other women/ … /but I think it was a bit strange. After six months you should recover somewhat after the delivery, but it took so long.

Restraining also came in the form of poor quality of sleep. This could either be due to difficulties to relax and fall asleep, or sleep being interrupted, because of pain. As all the women in this study were mothers of small children during the vitamin D treatment this meant that in addition to being disturbed by pain and immobility, sleep was also sometimes affected and interrupted by the child/children. In a combination, this could lead to a negative spiral of impaired sleep. It could end up being a compelling and “unnatural” need for sleep during the day while suffering from LLVD, as Hanna said: *Sometimes I slept during the day, and in the evening as well … but was also very tired afterwards. It´s not normal. You should be alert during the day, and sleep at night*. The necessity of sleeping during the day sometimes made the women feel incapable of caring for their children, and not being good mothers. One woman described how she was unable to take care of her children, or provide them with food due an overwhelming need to sleep:

Sometimes I feel I don´t want to see them [the children], since I´m so tired when they come and wake me, I say ”go away” I want to lie down, I want to sleep … you shouldn´t do that to your own children. (Hanna)

This meant that she was unable to cook for her children, and that she had to ask her husband for help.

The need of also sleeping during the day and needing to use every opportunity to take a nap, for example, by following the sleeping rhythm of their children, had the result that daily life revolved around the need to sleep, and finding ways to sleep during the day. It took all energy and restricted the possibility of being active and getting out; it was the antithesis of life itself.

### Recaptured vitality

The physical and emotional capacity increased after vitamin D treatment, an increased physical strength, capacity and alertness that enabled most of them to be more active.

This ability to set goals for the everyday life and being outdoors more often were valued highly. The enjoyment of physical activity and the ability to exercise became better and better for most of the women during vitamin D treatment. One woman described this:

I couldn´t train, but can now. I want to train. I began at first with five minutes, then ten, then 15 minutes, 20 minutes; I spend more time/ … /I feel I´m getting better and better … day by day. (Emma)

For some, increased level of activity could though have a back side as it could involve a feeling of stress related to less time to relax. One woman even experienced that her feeling of tiredness became worse despite continuous treatment with vitamin D, since she increasingly had to sleep during the day. She described a cycle of continuous tiredness:

Even if I take vitamin D tablets daily I feel, unfortunately, that I´ve gotten worse/ … /I just want to lie down. When I´ve eaten breakfast I just want to go lie down. (Stella)

The pain that was felt during LLVD could be related to different times during the day, as bedtime, or presenting itself at special body parts like ribcage, arms, elbows, wrists or legs. After receiving vitamin D treatment, the pain generally diminished although the experienced effect varied. For some the effect was significant. A large variety of over-the-counter medicines (paracetamol and COX inhibitors) to relieve pain and non-pharmacological treatment as sitting in the sauna, and contact with a variety of healthcare providers did not help. Vitamin D was what finally helped:

I went to a naprapath at work, because I had pain here [points to her rib cage]/ … /it didn´t help/ … /I had a lot of pain before, even if I went to the physiotherapist, and ah, I took a sauna. Perhaps it´s muscle pain or something that hurts. But now, no! Nothing! (Hanna)

The women told of how they slept better after receiving vitamin D treatment, for example, in terms of falling asleep earlier related to no longer being disturbed by backache:

When I went to bed I didn´t fall asleep right away. I had to wait maybe an hour/ … /my back ached. Now I fall asleep in ten minutes. I sleep maybe an hour longer now. Before I slept six hours at the most, but now maybe seven hours. (Emma)

Some of the women also told how they now needed less sleep because they felt more alert and awake. They had time for activities because they now could stay awake and do things while the children slept.

### The need for continuity in medication

The effect of vitamin D treatment was not immediate. The women found it took at least a month to notice any difference.

The women described occasional breaks in their vitamin D treatment. Reasons varied. Either they simply forgot or lacked a renewed prescription when needed. The women pausing medication experienced symptom recurrence, in term of tiredness or pain:

When I haven´t taken them [the vitamin D tablets] for a while, perhaps a week or two, my pills were finished … and the prescription wasn´t renewed. I began to notice the pain returning, that is, the pain I had before treatment came back … (Rosa)

Some were sceptical of and having an aversion towards taking the medication in general or due to side effects they believed was caused by vitamin D:

I don´t always take the vitamin D pills … I don´t like them … they make me fat … I`m hungry all the time … (Emma)

## Discussion

### Result and discussion

Results showed that the women experienced crippling ill-health with pain, weakness, tiredness, and sleep deprivation, during a period of low levels of vitamin D. They told of how they had not previously received vitamin D for vitamin D deficiency, but as first or second-generation non-western immigrant women, they may have had LLVD for several years before treatment (Bergström et al., 2014; Erkal et al., 2006; Kalliokoski et al., 2013, 2016).

The women expressed a paralyzing physical weakness that restricted their daily lives before vitamin D treatment. This weakness limited activities and made it difficult to perform even simple and ordinary activities. The women described increased physical strength during vitamin D treatment, which enabled them to be more active and get more exercise. The results from our qualitative study capture nuances that complements the findings of increased muscle strength in the previous quantitative study (Englund et al., 2017) as well as in other studies (Erkal et al., 2006; Kalliokoski et al., 2013, 2016; Stockton et al., 2011). In our study, the relieved pain enabled increased levels of activity, which could also lead to further experiences of tiredness which were, however, not paralyzing, and thus differed from the tiredness they felt from LLVD.

All women in this study described experiences of pain while living with LLVD, which affected sleep and eventually meant poor sleep quality. They also described benefits of relieved pain during the vitamin D treatment, allowing a better quality of sleep at night, and reduced need for daily naps. One woman felt that the vitamin D treatment did not decrease her need to sleep daytime. McCarty et al. (2012) argue that the relationship between sleep disturbance and vitamin D deficiency is complex, requiring further investigation. A recently published meta-analysis shows that LLVD significantly increases the risk of poor sleep quality, short sleep duration, and sleepiness (Gao et al., 2018). It has also been discussed whether vitamin D plays a role in pain relief among patients with vitamin D deficiency (Kragstrup, 2011). In our study, sleep (disturbance) and (diminished) pain were described as interconnected. An interconnectedness that has also been found among patients with a wide variety of other illnesses, and might even be inseparable to a degree where one symptom will increase when the other symptom increase (Finan, Goodin, & Smith, 2013; Wei, Blanken, & Van Someren, 2018).

The women talked about the many facets of ill-health while living with LLVD that became an antithesis to health and living the kind of active and responsible family life they expected, like being a good mother. The findings resonate well with views on health as “silent” and that it is through ill-health that one becomes aware of the body and its limitations (Leder, 1990). This can be important to keep in mind in discussions with women about their health concerns and decisions (Jenkinson, Kruske, & Kildea, 2017; Thom et al., 2016) and when explaining to women receiving vitamin D treatment why continuity in medication is important. The finding may also be of interest in discussions on placebo effects (Finniss, Kaptchuk, Miller, & Benedetti, 2010). On one hand, it may be argued that the women’s experiences of ill-health were related to the postpartum period rather than LLVD, and that the improvements resulted from psychological expectations on their vitamin D treatment rather than normalized levels. On the other hand, the women told that it took time to feel any improvement after starting their vitamin D treatment and that they discovered that their problems came back when interrupting the treatment. This could be an argument against explaining their experiences of ill-health only by the fact that they were all in a postpartum period and/or had low haemoglobin levels and a counterargument for considering their improvements only as a placebo effect.

An important finding in our study is that the women’s experiences of living with LLVD were “a blend” of family life with small children, work, pain, and tiredness in a vicious circle. It was difficult for them to assess whether their experiences were a normal part of life for a woman after childbirth. They told about experiences of ill health, often in terms of absent health (such as lacking the strength to run for the bus, or living an active life). It was thus difficult for them to express their ill-health in terms of particular “symptoms”. The women had diverse multicultural backgrounds and multi-cultural variations in experience of and verbalising symptoms are well known and have been found among patients with other diseases (Kwok, 2014; Mossey, 2011; Peacock & Patel, 2008; Shavers, Bakos, & Sheppard, 2010; Wei et al., 2018).

This is important knowledge for healthcare professionals to bear in mind when meeting women who are mothers of small children with diffuse physical tiredness and pain and calls for a broadened and cultural sensitive and safe care in a world with increasing migration (Williamson & Harrison, 2010; Woods, 2010).

### Methodological considerations and trustworthiness

n this study we explored health and ill-health in daily life among non-western immigrant women living with LLVD undergoing vitamin D treatment and reaching normalized levels after childbirth, using interviews to encourage descriptions in their own words, from their perspective. The qualitative design of the study contributed to understand the worlds of the patients, which is important (Rowe & McAllister, 2002) and can support a reflexive approach amongst healthcare providers (Jenkinson et al., 2017). The women were recruited from, and complementing, an intervention study examining vitamin D treatment after childbirth. Our sample consisted of women reaching normalized levels after one year of treatment. It may be argued that is not surprising that the women described benefits from their vitamin D treatment. Our study contributes with knowledge about how vitamin D deficiency and normalized levels after childbirth intermingle with everyday life. However, our sample is too small to draw any generalizable conclusions.

While our sample consists of immigrant women, we do not suggest that Vitamin D deficiency during post-partum is limited to this group of women. Further research might benefit from larger samples also including women not reaching normalized vitamin D levels, and a prospective and longitudinal design where participants are followed alongside their treatment trajectory.

The study was conducted following swedish regulations regarding research ethics on human subjects. Respect for enrolled subjects was met since all women were fully informed that participation was completely voluntary (three women declined participation). They were also informed that they could withdraw from the study at any moment without consequences for their clinical care. All women were given the possibility to choose interview location and time point; some women preferred to be interviewed at home, others preferred the interviewer’s workplace. Confidentiality was secured throughout the research process by excluding all information that could lead to identification during transcription, sample description and in the presentation of results.

The varied expertise and disciplinary background among the members of the research group provided openness to the nuances in the women’s descriptions, which enhanced credibility (Graneheim & Lundman, 2004).

### Concluding remarks

The study provides knowledge about the difficulties for women to distinguish precise symptoms that could be unequivocally connected to low levels of vitamin D. The women’s experiences of ill-health during the period of LLVD were described as entangled with daily life as mothers of small children. Lack of sleep is common and a part of life after childbirth, and pain can be associated with the postpartum period (McGovern et al., 2007; To & Wong, 2003) as well as other conditions such as fibromyalgia (Wepner et al., 2014), or symphysiolysis (Kanakanis, Roberts, & Giannoudis, 2011). Maternal fatigue and depression postpartum can be hard to distinguish and give experiences of exhaustion (Giallo, Gartland, Woolhouse, & Brown, 2015). Considering the limitations of this study, our findings indicate that it may be important for healthcare professionals to also have low levels of vitamin D in mind when screening for haemoglobin levels and when encountering immigrant women with small children describing experiences of pain, physical weakness, tiredness, poor quality of sleep, and the necessity to sleep during the day time. It may be important to ask these women about daily activities as a complement to questions about specific symptoms. Also, our study illuminates the importance of explaining that the effects of vitamin D treatment can be delayed, rather than immediate, and that continuity in medication is important. Future studies could investigate whether “physical weakness” and “muscle fatigue” are similar or different phenomena using a mixed methods design.
